# Epidemiology and microscopic diagnosis of tuberculosis in pigs and small ruminants slaughtered at Bobo-Dioulasso abattoir, Burkina Faso

**DOI:** 10.4102/ojvr.v88i1.1908

**Published:** 2021-12-03

**Authors:** Adama Sanou, Amadou Dicko, Kadiatou R. Sow, Arthur Djibougou, Antoinette Kabore, Bassirou Diarra, Arsène K. Ouedraogo, Dezemon Zingue, Moumini Nouctara, Zekiba Tarnagda

**Affiliations:** 1 Department of Biomedical, Centre MURAZ, Bobo-Dioulasso, Burkina Faso; 2 Training and Research Unit in Science and Technology, Nazi BONI University, Bobo-Dioulasso, Burkina Faso; 3 Department of Biomedical Sciences, Centre MURAZ, Bobo Dioulasso, Burkina Faso; 4 Faculty of Medicine and Dentistry (FMOS), University of Sciences, Techniques and Technologies of Bamako (USTTB), Bamako, Mali; 5 University of Sciences, Techniques and Technologies of Bamako, Bamako, Mali

**Keywords:** tuberculosis, small ruminants, pigs, routine inspection, Bobo-Dioulasso

## Abstract

Bovine tuberculosis (bTB) is a zoonotic, infectious, chronic and contagious disease, caused by *Mycobacterium bovis* that mainly affects cattle. This pathology has a negative impact on animals and animal products trade. Unfortunately, in Burkina Faso where agriculture and livestock sectors represent around 80% of the socio-economic activities, the real situation of the disease is not well known especially in small ruminants and swine. Thus, our study focused on both the epidemiology and the microbiological diagnosis of tuberculosis (TB) in small ruminants and pigs slaughtered at Bobo-Dioulasso abattoir. A prospective study was conducted between August 2017 and December 2017. Epidemiological data collection was performed during routine meat inspection; moreover, samples were taken and transported to the Bacteriology laboratory of Centre Muraz for microbiological analyses. This diagnosis consisted in search of Acid Fast Bacilli (AFB) using the hot Ziehl–Neelsen staining. Out of a total of 14 648 small ruminants and 2430 pigs slaughtered during the study period, 156 and 17 had lesions suggestive of bTB with prevalence of 1.07% and 0.7%, respectively. Females and those between 2 and 4 years old were mainly infected. The most affected organs were: lungs, liver, spleen and lymph nodes. Finally, microscopy revealed 43.35% (75/173) of positive cases for AFB. These results confirm the presence of bTB in small ruminants and pigs in Burkina Faso. Efforts must still be made in the fight against this zoonosis in order to limit its economic and public health impacts.

## Introduction

Livestock plays a central role in the economy of the Sahel part of Africa, which is illustrated as an excellent region for animal breeding. It is indeed one of the main economic activities on which the regional populations are dependent as a source of food and monetary income (CSAO/OCDE 2008). This sector is unfortunately under constant pressure of various parasitic, viral and bacterial pathologies. Amongst these, bovine tuberculosis (bTB) is one of the most difficult to control because of its insidious and chronic nature.

Bovine tuberculosis is a bacterial infectious disease caused by *Mycobacterium bovis* (*M. bovis*), whose main victims are not only domestic cattle but it also affects other animals (domestic and wild) and human (Cosivi et al. 1995; CSAO/OCDE 2008). It is considered a major disease for international trade of live animals and animal products, and is on the list of notifiable diseases of the World Organisation for Animal Health (OIE). Consequently, several developed countries have implemented campaigns to eliminate *M. bovis* from the cattle herd or at least to control its spread. The success of these control programmes has often been mitigated. Indeed, the fact that *M. bovis* infects a wide host range can complicate attempts to control or eradicate the disease in cattle. However, with multiple efforts to control it, the disease is rare in developed countries. Also, the introduction of pasteurisation considerably reduced public health risks in many countries, but the disease continues to cause production losses where it is poorly controlled.

Regrettably, diseases although eradicated from parts of Europe such as foot and mouth disease, rabies, bTB, remain enzootic in many developing countries. In Africa, in 1998 only seven countries (Algeria, Burkina Faso, Cameroon, Morocco, Namibia, South Africa, and Zambia) out of 55 had a bTB control programme in cattle herds, using tuberculin tests and post-mortem inspection for the surveillance of this disease (Cosivi et al. 1998). Currently, programmes are implemented in most African countries but remain ineffective because of not only the transhumance movements of animals from areas where the measures are not applied at all but also the lack of synergy between countries involved in the fight.

In the Sahel, more precisely in Burkina Faso, small ruminants and cattle breeding is practiced extensively and depends mostly on the availability of natural grazing and water. Their availability or abundance are linked to the annual rainfall, which is very often insufficient, thus forcing breeders and their livestock to move from arid regions to more humid ones. In addition, the existence of priority enzootic diseases, such as contagious bovine pleuropneumonia (CBPP), foot-and-mouth disease, which decimate among cattle, and the lack of means and the insidious nature of bTB have led to continuous neglect of this disease in the country. The government meat inspection regulations however, provide for regular checks of animal carcasses before consumption. Thus, the only information available most often comes from suspicions during routine meat inspection at slaughterhouses and slaughter areas. Nevertheless, the existence of bTB has been known for a very long time in this country, where the highest rates from all French West African countries were observed in slaughterhouses (Regnoult 1963). Nonetheless, several studies on bTB have only concentrated on cattle, and there are few data available on small ruminants and pigs despite their numerical and economic importance.

Thus, the study aims to provide epidemiological and microbiological data on bTB in small ruminants and pigs slaughtered at the Bobo-Dioulasso abattoir to contribute to public health and bTB control improvement in the country.

## Material and methods

### Type, period and study sites

This was a prospective cross-sectional study that took place at Bobo-Dioulasso abattoir (Hauts Bassins region, Burkina Faso) between August 2017 and December 2017. The study settings also included Centre MURAZ, where the laboratory analyses were carried out. In addition, the animals (small ruminants and pigs) studied potentially originate from different parts of the country.

### Sampling

We did an exhaustive sampling. Our study focused on cases of organ seizures for suspected TB during the study period. The samples consisted of tissues from suspected organs such as lymph nodes (prescapular, pulmonary, hepatic, mandibular, retropharyngeal …), lungs, spleen, liver, udders …, following routine meat inspection at the slaughterhouse.

### Data collection

Data collection was carried out using a sheet developed for this study. The data collected were epidemiological and bio-clinical information, in particular the registration number of the suspected animal, the type of seizure, age, sex and anatomo-pathological aspects of the carcass (carcass appearance, lesions location). This information was collected through observation by veterinary technicians during meat inspection.

## Study methods

### Sample processing

#### Sampling and transport

At Bobo-Dioulasso Abattoir, animals are mostly slaughtered daily from midnight to 6 am and during the day to ensure permanence. The carcasses were inspected by veterinary doctors and officers. Pieces of organs from suspect animals were transported at 4 °C to Centre MURAZ for microbiological analyses. Three bio-clinical samples per suspected animal were taken to increase the probability of bTB detection.

#### Sample pre-processing

Pre-treatment consistent of samples decontamination organ pieces and grinding. The samples taken were previously cleaned with sterile distilled water before treatment using the Saeng and Costil method (Delafosse, Traore & Kone 1995). For the pre-treatment itself, the suspected piece was first deposited in a sterile Petri dish and then cut with a scissor in a sterile manner. Secondly, the tissue fragments were finely ground in a sterile mortar in presence of sterile sea sand. Then 10 mL of sterile distilled water was added to the homogenate and the mixture was collected in a sterile 50 mL Falcon tube.

Finally, after 5 min decanting, a 2 mL volume of supernatant was recovered and placed in another sterile 50 mL Falcon tube, and then processed according to Petroff method (Palomino & Portaels 1998) for decontamination in agreement with the following procedure:

- Step 1: A total of 10 mL of NaOH were added to the 2 mL of recovered supernatant and the mixture was vigorously mixed with a vortex for 30 s, then for 20 min on the Kahn agitator.
- Step 2: The suspension was then completed with sterile distilled water until an average volume of 45 mL and mixed on a vortex for 30 s.
- Step 3: Subsequently, the suspension was centrifuged for the first time at 3000 revolutions per minute (rpm) for 20 min. Step 3 was repeated 3 times.
- Step 4: After removal of the supernatant, a volume of 1.5 mL of sterile distilled water was added to the pellet and the tube was vigorously vortexed to until obtained an homogenous mixture, ready for microbiological analysis.

### Microbiological analysis methods

One part of the pellet was used for microscopical analysis using Ziehl–Neelsen staining so as to detect the presence of Acid Fast Bacilli (AFB). In order to have optimal conditions, all the slides to be used must be sterilised with a fire source (typically a spirit lamp) applying the heat on both faces for a few seconds. A certain volume of the sample is extracted and spread on the slide making a thin uniform barely transparent smear with a sterile bacteriological sampling loop. Once this is done, the spread is fixed with a little heat from the spirit lamp, applied (only) under the slide before undergoing staining using the hot Ziehl–Neelsen method.

## Data analysis

A double entry was used to record data using Sphinx Plus2 V5 software where appropriate, descriptive parameters such as sums, percentages and fractions were then computed. Statistical analysis was performed using Epi info version 7.2.2.6. Logistic regression was used to assess the relationship between lab results and animal characteristics. The *p* < 0.05 value was considered statistically significant.

### Results

#### Frequencies of suspected tuberculosis lesions

Out of 14 648 total small ruminants and 2430 pigs slaughtered during the study period, 156 and 17 had TB suspected lesions giving prevalence of 1.07% and 0.70%, respectively, and an overall prevalence of 1.01% (see Table 1).

**TABLE 1:** Frequency of suspicious lesions in small ruminants and pigs slaughtered at the Bobo-Dioulasso abattoir from August to December 2017.

Animal species	*N*	*n*	%
Small ruminants	14 648	156	1.07
Swine	2,30	17	0.70
**Total**	**17 078**	**173**	**1.01**

*N*, number of carcasses inspected during the study period; *n*, number of carcasses with suspected TB lesions; %, prevalence of suspected cases.

#### Distribution of tuberculosis suspect animals by age and sex

**FIGURE 1:**
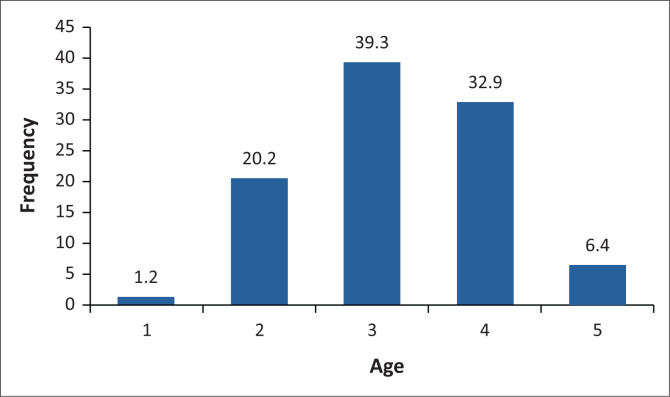
Repartition of tuberculosis suspected animals by age.

Of the 173 suspected bTB carcasses, 141 (81.5%) were females and 32 (18.5%) were males, that is, a sex ratio of 0.23 (32/141) (see Table 2). The age of the animals were between 1 year and 5 years as shown in Figure 1.

**TABLE 2:** Sex ratio in small ruminants and pigs suspected of tuberculosis at the abattoir from August 2017 to December 2017.

Animal species	M	F	Sex ratio
Sheep	22	91	0.24
Goats	7	36	0.20
Swine	3	14	0.21
**Total**	**32**	**141**	**0.23**

M, male; F, female.

#### Location of Bovine tuberculosis lesions

**FIGURE 2:**
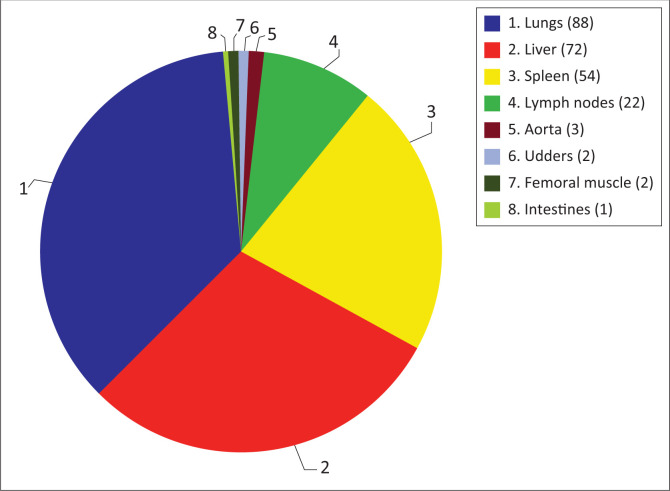
Distribution of suspicious Bovine tuberculosis lesions by organ in small ruminants (sheep and goats) and pigs at Bobo-Dioulasso slaughterhouse.

Of the 173 suspected animals, 95.38% presented localised lesions to some organs (lungs, liver, spleen, lymph nodes, …) against 4.62% with generalised lesions throughout the carcass. The most affected organ was the lung with a proportion of 36.07%. However, there were sub-populations specificities because the liver was the most affected organ for pigs. For affected organs and the relative numbers refer to Figure 2.

#### Proportion of suspected Bovine tuberculosis cases based on microscopic examination

Amongst the 173 carcasses with suspicious lesions, microscopy revealed the presence of bTB in 75 (43.35%) samples. Table 3 shows the microscopy positive results by animal species.

**TABLE 3:** Microscopy results of the different suspected carcasses (sheep, goats and pigs).

Animal species	Positive	Percentage
Sheep	52	69.33
Goats	15	20.00
Swine	8	10.67
**Total**	**75**	**100.00**

#### Risk factor analyses

**FIGURE 3:**
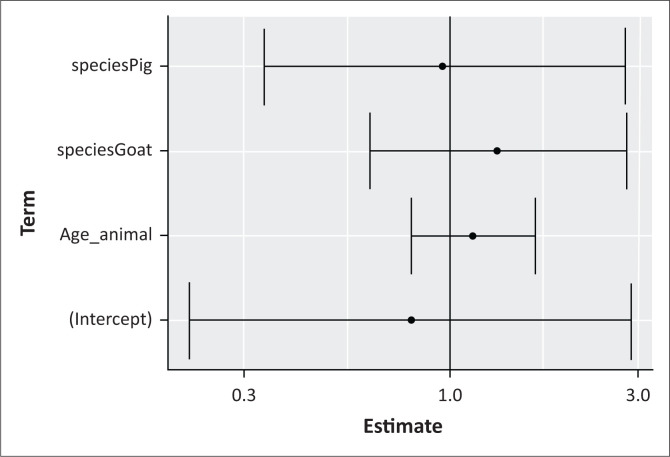
Multivariate analysis of laboratory results according to animal characteristics.

The results of multivariate analysis of laboratory results according to animal characteristics are shown in Figure 3. This shows that the infection is neither related to age nor to the animal species.

## Discussion

The objective of this study was to explore the full breadth of bTB in small ruminants and swine populations in Burkina Faso, suspecting its presence based on its frequency in cattle slaughtered and routinely inspected in Burkina Faso slaughterhouses. The present study indicates the existence of bTB in Burkina Faso with a proportion of 1.01% of lesions suggestive of this pathology in small ruminants and swine inspected. The presence of bTB in small ruminants and pigs is not surprising; indeed, evidence of bTB existence dates back to the colonial period based on single cervical intradermal test in cattle (Sere 1966). In addition, bTB cases have been reported regularly in cattle in many sites of this country and specifically at Bobo-Dioulasso slaughterhouse based on routine meat inspection (Delafosse et al. 1995; Tarnagda et al. 2014; Vekemans et al. 1999), sometimes with probable transmission between animals (Sanou et al. 2014). Regular contacts between different animal species on breeding areas may then explain the circulation of the bacillus and persistence of the disease between and within the different animal sub-populations, respectively (Gelalcha, Zewude & Ameni 2019).

In the present study, the prevalence of the disease was higher in small ruminants compared with pigs, with respective prevalence of 1.07% and 0.70%. Our results were higher than the 0.03% found in goats in Nigeria in 2016 (Danbirni et al. 2016) and 0.15% and 0.07%, respectively, in sheep and goats found in Togo 8 years earlier (Kulo & Seme 2008). The rates observed elsewhere in Africa are higher than those observed in this study. Indeed, the prevalence of bTB was 6.03% in goats in Algeria in 2011 (Naima et al. 2011), and 5.80% in Ethiopia in pigs in 2013 (Arega, Conraths & Ameni 2013). Higher prevalence has also been reported in Europe. Indeed, it was 10.00% in goats and 3.20% in sheep in Spain in 2018 (Vidal et al. 2018) and 4.90% in pigs in Italy the same year (Amato et al. 2018). The present study in addition to confirming the bTB existence in small ruminants and swine populations in Burkina Faso, suggests a slight decrease in its prevalence in pigs compared with the data reported (2.150%) 50 years ago (Gidel et al. 1969). The difference in prevalence between our study and those of these authors could be explained by several factors related mainly to farming practices and conditions. The production system with the differences in animal breeding, animal populations density, aeration conditions and introduction of new animals are all factors that influence bTB prevalence. Thus, within the same geographic region, the prevalence of bTB can vary significantly from one area to another.

Regarding parameters such as age and sex of slaughtered animals, they are not routinely collected by veterinary officers. The data collected during the present study were only related to suspected bTB cases. They show that suspected animals were mainly females and between 2 years and 4 years of age even if sex, age are not significantly associated with the prevalence. Previous studies have shown a link between sex and bTB occurrence (Lamireou 2014). These studies found that the disease incidence and seizures were significantly higher in females than males. This could probably be because of hormonal influence, gestation, parturition and lactation, which weaken female’s immune system and thus increase her vulnerability to diseases (Kudi et al. 1997). In addition, more females than males are generally slaughtered routinely because the demand for males is affected by religious and cultural events such as Christmas, Muslim holidays and baptisms and marriages. Most of males are kept for slaughter during these periods. As far as age is concerned, it is not generally a risk factor. When it is associated with environmental factors production system (intensive breeding) which favour bacteria circulation, the probability of bTB germ infection increases, thus the prevalence can be higher in older animals (Boukary et al. 2011). However, age can also lead to a physiological decrease in immunity and therefore constitutes an immunosuppressive factor.

Regarding the lesions extent that caused carcass seizures during routine inspection, most were located on a given organ. Lung damage is the leading cause of bTB seizure in sub-Saharan Africa (Kulo & Seme 2008; Naima et al. 2011). Our study confirmed that lungs represent the main localisation for mycobacteria with a frequency of 36.07%. This is also explained by mycobacteria tropism, which infect the lungs first in most cases before spreading to other organs in accordance with air route as bTB’s main transmission mode (Ullah et al. 2019). These results are similar to those obtained in Sudan (Aljameel, Mohammed & Bakhiet 2017) However, other transmission routes such as buccopharyngeal also play a non-negligible role with more than 15% of cases. This can explain the percentage of other lesion types frequently encountered in slaughterhouses such as lymph node reactions, lesions in liver, spleen, etc. The same observation has been made by other authors (Amato et al. 2018). These tissues are generally amongst the most affected during routine examination carried out by veterinary inspectors, and thus may contain caseous nodules, usually reflecting chronic bTB infection (Amato et al. 2018). The liver was the most involved organ in swine, and this may be because of confusion between bTB lesions and those of porcine cysticercosis, which is very common in the study area. On the economic level, seizures, especially total ones (0.047%), still constitute a considerable loss for butchers because they receive no compensation. As a result, some pet owners strongly protest and express their outrage for seizure decisions. This total seizure measure would lead to increase in clandestine slaughter as reported by veterinary services. This practice promotes bTB transmission to humans, mainly by handling infected carcasses in poor hygienic conditions (Sa’idu et al. 2015).

The proportion of suspected bTB positive lesions in microscopy was 43.35%. Despite the microscopic examination low sensitivity, our study found a prevalence above the 13.33% found in Algeria in goats (Naima et al. 2011) and 10.89% found in Democratic Republic of Congo (Luboya et al. 2017). However, the prevalence obtained in the present study was below 76.1% found in cattle in Sudan (Aljameel et al. 2015). As TB microscopy is most often focused on diagnosis of human cases, the AFB search in animal samples requires patience and special attention because of the morphological dissimilarities between the different types of TB bacilli, human and bovine.

## Conclusion

This study showed the bTB occurrence in small ruminants and pigs slaughtered at Bobo-Dioulasso abattoir with a prevalence of 1.01% of suspicious lesions. The study found that lungs damage was the leading cause of bTB seizure at Bobo-Dioulasso slaughterhouse. Also, the female and old animals were the most infected. Additional control efforts are required for more effective control of this zoonosis in Burkina Faso.
